# Sarcopenia associated with increased risk of comorbidities among individuals with chronic kidney disease: insights from a prospective cohort

**DOI:** 10.3389/fpubh.2025.1672639

**Published:** 2025-12-16

**Authors:** Yuanyi Feng, Dan Li, Yifu Weng, Chenfang Song, Zhenhe Huang, Hualiang Lin

**Affiliations:** 1 Department of Geriatrics, Affiliated Nanshan Hospital of Shenzhen University, Shenzhen, China; 2 Department of Epidemiology, School of Public Health, Sun Yat-sen University, Guangzhou, China; 3 Department of Emergency Medicine, Affiliated Nanshan Hospital of Shenzhen University, Shenzhen, China

**Keywords:** sarcopenia, chronic kidney disease, handgrip strength, comorbidities, UK Biobank

## Abstract

**Background:**

Although sarcopenia is associated with an increased risk of chronic kidney disease (CKD), its potential associations with subsequent comorbidities among patients with CKD remain unknown.

**Methods:**

This prospective study included 19,502 participants with CKD, defined as an estimated glomerular filtration rate less than 60 mL/min/1.73 m^2^. Sarcopenia is characterized according to muscle strength, muscle mass, and physical performance; low muscle strength, commonly measured using handgrip strength, is considered the most important indicator. The study outcome was post-CKD comorbidity. Multivariable Cox proportional hazards models were used to analyze the effect of sarcopenia on comorbidities. Population attributable fractions (PAFs) and potential impact fractions (PIFs) were used to quantify the population-level burden and potential benefit of improving handgrip strength.

**Results:**

During a median follow-up of 10.6 years, 5,374 participants developed one comorbidity, 2,972 developed two comorbidities, and 2,434 developed three or more comorbidities. Sarcopenia was associated with a graded increase in multimorbidity risk (approximately 12, 24, and 33% higher for one, two, and three or more comorbidities, respectively, versus non-sarcopenia). Lower handgrip strength exhibited a clear exposure–response, and the lowest tertiles was associated with the greatest risk across outcomes. Among individual comorbid diseases, the strongest association was observed with osteoporosis. PAFs indicated that 5.21, 13.72, and 23.46% of cases involving one, two, or three or more comorbidities, respectively, were attributable to sarcopenia. Analysis of PIFs indicated that improving handgrip strength throughout the population (i.e., shifting lower to higher tertiles) could reduce the burden of one, two, and three or more comorbidities by approximately 12, 16, and 22%, respectively.

**Conclusion:**

The results indicate that sarcopenia, especially low handgrip strength, increases the risk of developing comorbidities, particularly osteoporosis, among patients with CKD. Quantification of PAFs and PIFs underscores the clinical and public health potential of muscle strength assessment and strength-preserving interventions to mitigate the CKD-associated comorbidity burden.

## 1 Introduction

Chronic kidney disease (CKD) affects approximately 10% of the global population (1) and is associated with poor outcomes and substantial healthcare expenditures, which are mainly due to the high burden of post-CKD comorbidities (2, 3). Evidence suggests that the 10-year renal survival rate among patients with CKD decreases as the number of comorbidities increases (4), and patients with more comorbidities often experience more rapid disease progression (5). Despite advances in nephrology care, preventing or mitigating comorbidities subsequent to CKD remains a major clinical and public health challenge.

Sarcopenia, defined by a reduced muscle strength, low muscle mass, and impaired physical performance, is far more common in people with CKD than in the general population (6) and has been linked to adverse outcomes such as mortality (7, 8), cardiovascular complications (9), and progression to end-stage kidney disease (10). However, previous research involving patients with CKD has mainly examined single outcomes rather than the long-term accumulation of multiple comorbidities across organ systems (9, 10). In CKD, sarcopenia often reflects a systemic state of vulnerability that involves chronic inflammation, gut dysbiosis, disturbed mineral–bone metabolism, malnutrition, hormonal imbalance (including insulin resistance and hypogonadism), and oxidative stress (11–13). The involvement of these pathophysiological pathways suggests that sarcopenia may not only accompany CKD but also accelerate the development of multiple chronic conditions, including osteoporosis (14), cognitive impairment (15), anemia (15), respiratory infections (16), and diabetes (17), over time. However, whether sarcopenia at baseline predicts the longitudinal accumulation of comorbidities in patients with CKD has not been clarified in a large-scale prospective cohort.

To address this gap, we conducted a large prospective cohort study to evaluate the association between sarcopenia and comorbidities among patients with CKD. We investigated whether sarcopenia and low handgrip strength were associated with not only the incidence of individual new chronic conditions but also a comorbidity risk gradient, defined as the development of one, two, or three or more comorbidities after CKD. We further quantified the population attributable fractions (PAFs) and potential impact fractions (PIFs) to estimate the proportions of comorbidities that would be potentially preventable through hypothetical improvements in muscle strength. The findings will provide evidence to prioritize strategies for preventing post-CKD comorbidities.

## 2 Methods

### 2.1 Study design and participants

The UK Biobank is a large prospective cohort of more than 500,000 participants aged 37–73 years who were recruited from 22 centers across England, Wales, and Scotland during 2006–2010 (18). This study collected comprehensive data on participants’ socioeconomic and lifestyle factors, diet, disease status, and medication history using physical and biological measurements, oral interviews, and questionnaires. The UK Biobank study was approved by the North West Research Ethics Committee (06/MRE08/65).

**Figure 1:**
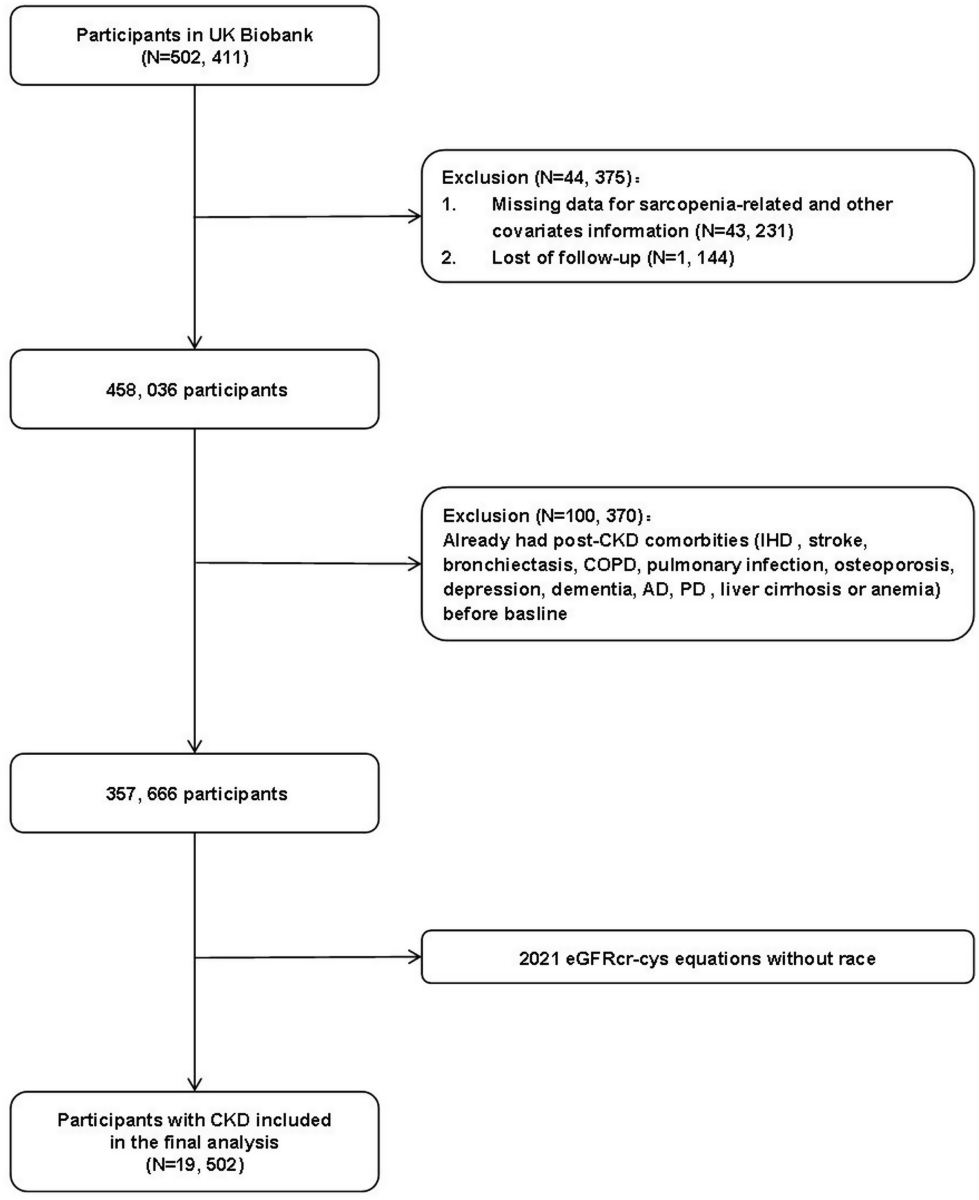
Flow chart of participant selection in this study.

For the current analysis, we excluded participants who had post-CKD comorbidities at baseline [ischemic heart disease (IHD), stroke, bronchiectasis, chronic obstructive pulmonary disease (COPD), pulmonary infection, osteoporosis, depression, dementia, Alzheimer’s disease (AD), Parkinson’s disease (PD), liver cirrhosis, or anemia; *n* = 100,370], had missing data on sarcopenia-related and other covariates [handgrip strength, appendicular lean mass index (ALMI), and gait speed; *n* = 43,231], or were lost to follow-up (*n* = 1,144) (Supplementary Figure 1). The final analysis included 19,502 participants (Figure 1), and their CKD diagnoses are detailed in the Supplementary material.

To enhance the accuracy of CKD diagnosis, eGFR was calculated using laboratory test values for serum creatinine and cystatin C and the 2021 eGFRcr-cys equations without race (19). The eGFR value and urine albumin-creatinine ratio (uACR) were then used to define CKD by eGFR <60 mL/min/1.73 m2 or uACR ≥3 mg/mmol (20). Additionally, individuals with a diagnosis of CKD [International Classification of Diseases Tenth Revision (ICD-10): N18] or proteinuria (ICD-10: R80.9) were considered to have CKD regardless of their eGFR and uACR values.

### 2.2 Sarcopenia assessment

Sarcopenia was diagnosed according to the EWGSOP2 criteria, which focus on the assessment of three dimensions: muscle mass, muscle strength, and physical performance (7).

We classified cases of sarcopenia (n = 1,040) into three types: (1) probable sarcopenia (*n* = 1,010), indicated by the presence of low muscle strength alone (grip strength < 27 kg for men, <16 kg for women) without low muscle mass and low physical performance; (2) confirmed sarcopenia (*n* = 23), confirmed by the presence of both low muscle strength and low muscle mass (ALMI < 7.0 kg/m^2^ for men, <5.5 kg/m^2^ for women) without low physical performance; and (3) severe sarcopenia (*n* = 7), characterized by the presence of low muscle strength, low muscle mass, and low physical performance, assessed based on a slow walking pace (<1.3 m/s or <3 miles per hour) (Supplementary Figure 1). However, as few participants had severe or confirmed sarcopenia (*n* = 30), they were grouped with those with probable sarcopenia in the analyses.

### 2.3 Comorbidity definitions

Post-CKD comorbidities were defined as chronic conditions (other than CKD) identified in the participants (21). We determined whether each participant had developed one, two, or three or more comorbidities. For ascertainment, we integrated electronic health records, ICD-10 coding, and self-reported data to ensure comprehensive classification. We assessed the occurrence of IHD, stroke, bronchiectasis, COPD, pulmonary infection, osteoporosis, depression, dementia, AD, PD, liver cirrhosis, and anemia at baseline and during follow-up. The full lists of ICD-10 codes used to define each condition are provided in the Supplementary material.

### 2.4 Covariates

We considered a series of important covariates that have been shown to be associated with either post-CKD comorbidities or sarcopenia. The demographics, socioeconomic factors, and lifestyle factors included for analysis were age at recruitment (continuous variable), sex, ethnicity (White and non-White), body mass index (BMI; underweight < 18.5 kg/m^2^, normal: 18.5–25.0 kg/m^2^, overweight: 25.0–30.0 kg/m^2^, and obesity ≥ 30.0 kg/m^2^), annual household income (>€100,000, €52,000–€100,000, €31,000–€51,999, €18,000–€30,999, <€18,000), educational qualifications [college or university degree, A/AS levels or equivalent, O levels/GCSEs or equivalent, CSEs or equivalent, NVQ/HND/HNC or equivalent, and other (e.g. nursing or teaching)], deprivation index, smoking status (never, previous, and current), alcohol intake (never, occasional, moderate, and heavy), physical activity (low, moderate, and high), and dietary diversity score (DDS) level (low, medium, and high). The DDS was calculated based on the intake of five primary food groups: grains, fruits, vegetables, dairy products, and sources of animal protein and protein alternatives. We categorized the DDS into three levels, low, medium, and high, for practical use in public health applications. The analysis also included clinical characteristics, namely hypertension and diabetes. Hypertension was defined based on a record of ICD-10 codes (I10–I15) and linked to self-reported data, hospital admissions, death registries, blood pressure measurements (systolic blood pressure ≥140 mmHg or diastolic blood pressure ≥90 mmHg), or any self-reported use of antihypertensive medications. Similarly, diabetes was defined based on ICD-10 codes (E10–E14), a glycated hemoglobin level ≥48 mmol/mol, or any use of antihyperglycemic medications at the time of cohort recruitment (22–24).

### 2.5 Statistical analysis

Baseline characteristics are presented as means (standard deviations) for continuous variables and counts (percentages) for categorical variables related to sarcopenia. Group differences were assessed using the *t*-test or *χ*^2^ test as appropriate.

Multivariable Cox proportional hazards regression models were used to examine the associations between sarcopenia and comorbidities in CKD patients. Cox models are appropriate in this context because they provide interpretable hazard ratios (HRs) for time-to-event outcomes (25). Probable sarcopenia, confirmed sarcopenia, and severe sarcopenia were pooled into a sarcopenia category for statistical modeling to avoid small-sample issues and increase the statistical power. We analyzed the associations between comorbidity and each of the three dimensions used to define sarcopenia (low muscle strength, low muscle mass, and low physical performance). We used two models: Model 1 (basic model) was adjusted for age and sex, while Model 2 (adjusted model) was further adjusted for BMI, ethnicity, education, income, deprivation index, physical activity, smoking status, alcohol intake, DDS, hypertension, and diabetes.

Following the EWGSOP2 criteria, we treated low muscle strength as the key determinant of sarcopenia and focused on handgrip strength. We categorized handgrip strength into tertiles, with the highest tertile as the reference (7, 26). To address potential non-linearity between handgrip strength and risk, we used penalized cubic splines within the Cox model (27). Splines allow data to capture exposure–response without assuming linearity, while a smoothing penalty limits overfitting and improves generalizability. Knots were chosen via cross-validation and placed evenly across the exposure range; linear constraints at the boundaries were used to stabilize tail estimates (28).

### 2.6 PAF and PIF analyses

We quantified the potential population impact of muscle strength using population attributable fractions (PAFs) and potential impact fractions (PIFs), which were derived from adjusted hazard ratios (29). PAFs were used to estimate the proportion of cases attributable to having the lowest handgrip strength level (30). PIFs were used to estimate the expected percentage reduction in cases under two scenarios (29): Scenario 1 represented a population-wide intervention approach, where individuals in the lowest tertile shifted to the middle tertile and those in the middle tertile shifted to the highest tertile. Scenario 2 represented a targeted intervention, where only individuals in the lowest tertile for each exposure shifted to the middle tertile.

### 2.7 Stratified and sensitivity analyses

Stratified analyses were conducted by sex (male and female), age (<65 years and ≥65 years), BMI (<25 kg/m^2^ and ≥25 kg/m^2^), physical activity (low and high/moderate), smoking status (never and previous/current), alcohol intake (never and occasional/moderate/heavy), and DDS (low and high/medium). To test the interaction between subgroup and sarcopenia, we performed likelihood ratio tests comparing models with and without multiplicative interaction terms.

Sensitivity analyses were performed to check the robustness of the results. The details are provided in the Supplementary material.

## 3 Results

### 3.1 Characteristics of the study participants

Among the 19,502 participants, 1,040 (5.3%) had sarcopenia, and the mean age was 61.5 years (standard deviation: 6.5 years). Compared with participants without sarcopenia, those with sarcopenia were older, more likely to be female, had a lower DDS, engaged in less vigorous physical activity, and were more likely to have hypertension and diabetes. The majority of participants with sarcopenia (approximately 89.6%) were White and had a low educational level (Table 1).

**Table 1:** Baseline characteristics of the participants.

Variables	Sarcopenic	Non-sarcopenic	Overall	*p*-value^a^
	Mean ± SD or *n* (%)			
No. of participants	1,040	18,462	19,502	
Age (years)	62.8 (5.9)	61.5 (6.5)	61.5 (6.5)	<0.001
Sex				<0.001
Female	589 (56.6)	9, 515 (51.5)	10, 104 (51.8)	
Male	451 (43.4)	8, 947 (48.5)	9, 398 (48.2)	
Ethnicity				<0.001
White	932 (89.6)	17, 435 (94.4)	18, 367 (94.2)	
Non-white	108 (10.4)	1, 027 (5.6)	1, 135 (5.8)	
BMI, kg/m^2^				<0.001
Underweight (<18.5)	4 (0.4)	32 (0.2)	36 (0.2)	
Normal (18.5–25.0)	212 (20.4)	3, 558 (19.3)	3, 770 (19.3)	
Overweight (25.0–30.0)	453 (43.6)	7, 947 (43.0)	8, 400 (43.1)	
Obese (≥30.0)	371 (35.7)	6, 925 (37.5)	7, 296 (37.4)	
Annual household income, €				<0.001
>100,000	14 (1.3)	334 (1.8)	348 (1.8)	
52,000–100,000	58 (5.6)	1, 761 (9.5)	1, 819 (9.3)	
31,000–51,999	126 (12.1)	3, 212 (17.4)	3, 338 (17.1)	
18,000–30,999	240 (23.1)	4, 532 (24.5)	4, 772 (24.5)	
<18,000	351 (33.8)	5, 223 (28.3)	5, 574 (28.6)	
Unknown	251 (24.1)	3, 400 (18.4)	3, 651 (18.7)	
Education qualifications				<0.001
College or university degree	192 (18.5)	4, 029 (21.8)	4, 221 (21.6)	
A levels/AS levels or equivalent	89 (8.6)	1, 619 (8.8)	1, 708 (8.8)	
O levels/GCSEs or equivalent	190 (18.3)	3, 978 (21.5)	4, 168 (21.4)	
CSEs or equivalent	40 (3.8)	802 (4.3)	842 (4.3)	
NVQ or HND or HNC or equivalent	73 (7.0)	1, 478 (8.0)	1, 551 (8.0)	
Others (e.g., nursing, teaching)	62 (6.0)	1, 135 (6.1)	1, 197 (6.1)	
None of the above	394 (37.9)	5, 421 (29.4)	5, 815 (29.8)	
Deprivation index	−0.7 (3.4)	−1.2 (3.1)	−1.2 (3.1)	<0.001
Smoking status				<0.001
Never	577 (55.5)	9, 340 (50.6)	9, 917 (50.9)	
Previous	361 (34.7)	7, 187 (38.9)	7, 548 (38.7)	
Current	102 (9.8)	1, 935 (10.5)	2, 037 (10.4)	
Alcohol intake				<0.001
Never	170 (16.3)	1, 975 (10.7)	2, 145 (11.0)	
Occasional	289 (27.8)	5, 029 (27.2)	5, 318 (27.3)	
Moderate	442 (42.5)	8, 060 (43.7)	8, 502 (43.6)	
Heavy	139 (13.4)	3, 398 (18.4)	3, 537 (18.1)	
Physical activity				<0.001
Low	232 (22.3)	4, 140 (22.4)	4, 372 (22.4)	
Moderate	479 (46.1)	7, 783 (42.2)	8, 262 (42.4)	
High	329 (31.6)	6, 539 (35.4)	6, 868 (35.2)	
DDS level				<0.001
Low	355 (34.1)	5, 984 (32.4)	6, 339 (32.5)	
Medium	670 (64.4)	12, 198 (66.1)	12, 868 (66.0)	
High	15 (1.4)	280 (1.5)	295 (1.5)	
Hypertension	712 (68.5)	12, 606 (68.3)	13, 318 (68.3)	<0.001
Diabetes	192 (18.5)	2, 777 (15.0)	2, 969 (15.2)	<0.001

SD, standard deviation; BMI, body mass index; DDS, dietary diversity.

a *p*-values were calculated by *t*-test, *χ*^2^ test or Fisher’s exact test, as appropriate (*p < 0.05).

### 3.2 Sarcopenia and post-CKD comorbidities

**Figure 2:**
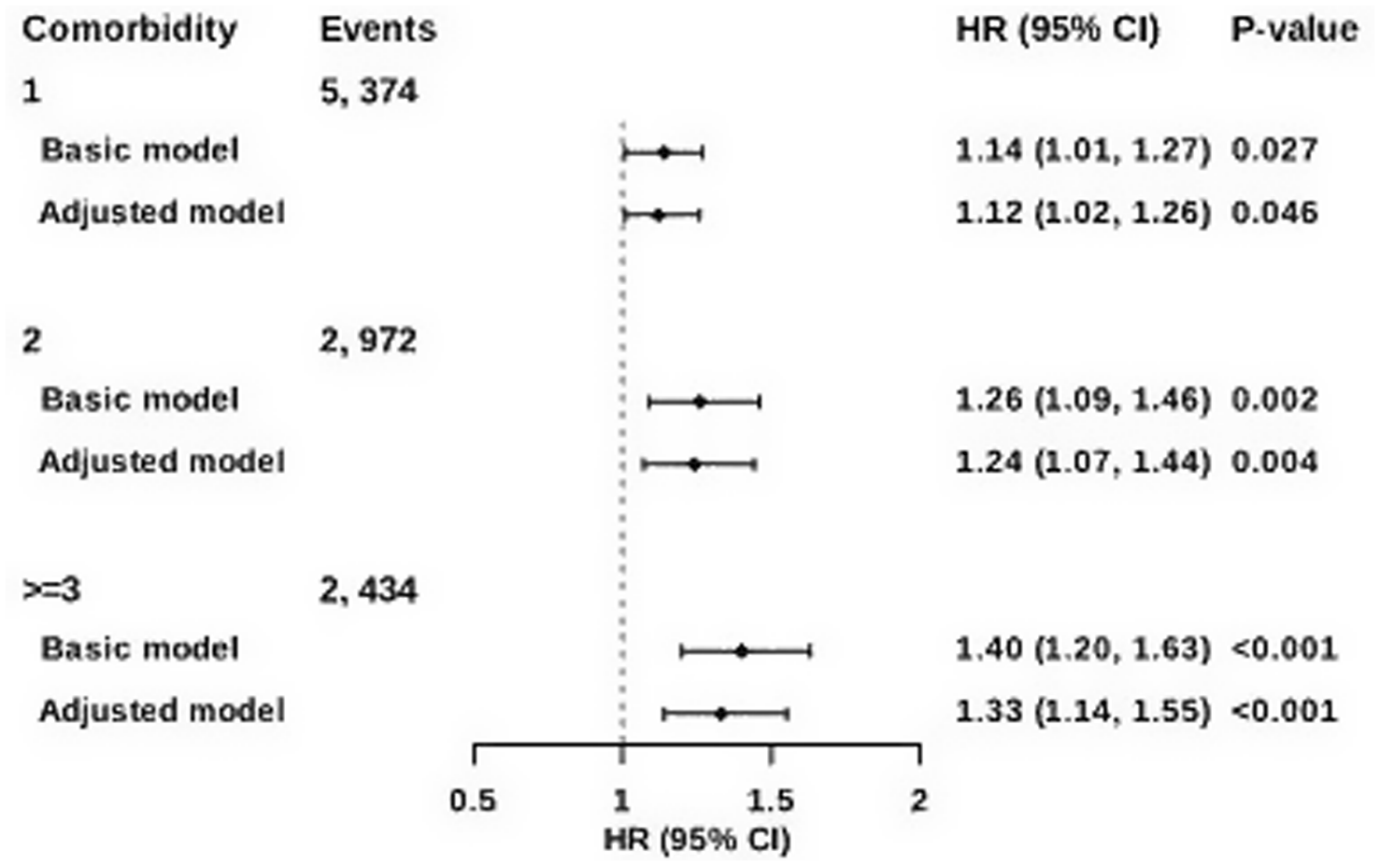
Association of sarcopenia with post-CKD comorbidities. Data are presented as hazard ratios (HRs) and 95% confidence interval (CI). Comorbidities included ischemic heart disease, stroke, bronchiectasia, COPD, pulmonary infection, osteoporosis, depression, dementia, AD, PD, liver cirrhosis and anemia. Basic model was adjusted for age and sex. Adjusted model was additionally adjusted for BMI, ethnicity, education, income, deprivation index, physical activity, smoking status, alcohol intake, DDS, hypertension and diabetes.

During a median follow-up of 10.6 years, 5,374 of 19,502 CKD participants (27.6%) developed one new comorbidity, 2,972 (15.2%) developed two, and 2,434 (12.5%) developed three or more comorbidities. In fully adjusted models, sarcopenia was associated with a 33% increase in the risk of progressing to three or more comorbidities [95% confidence interval (CI): 1.14, 1.55], with more modest increases for one (HR: 1.12; 95% CI: 1.02, 1.26) and two comorbidities (HR: 1.24; 95% CI: 1.07, 1.44) (Figure 2).

**Figure 3:**
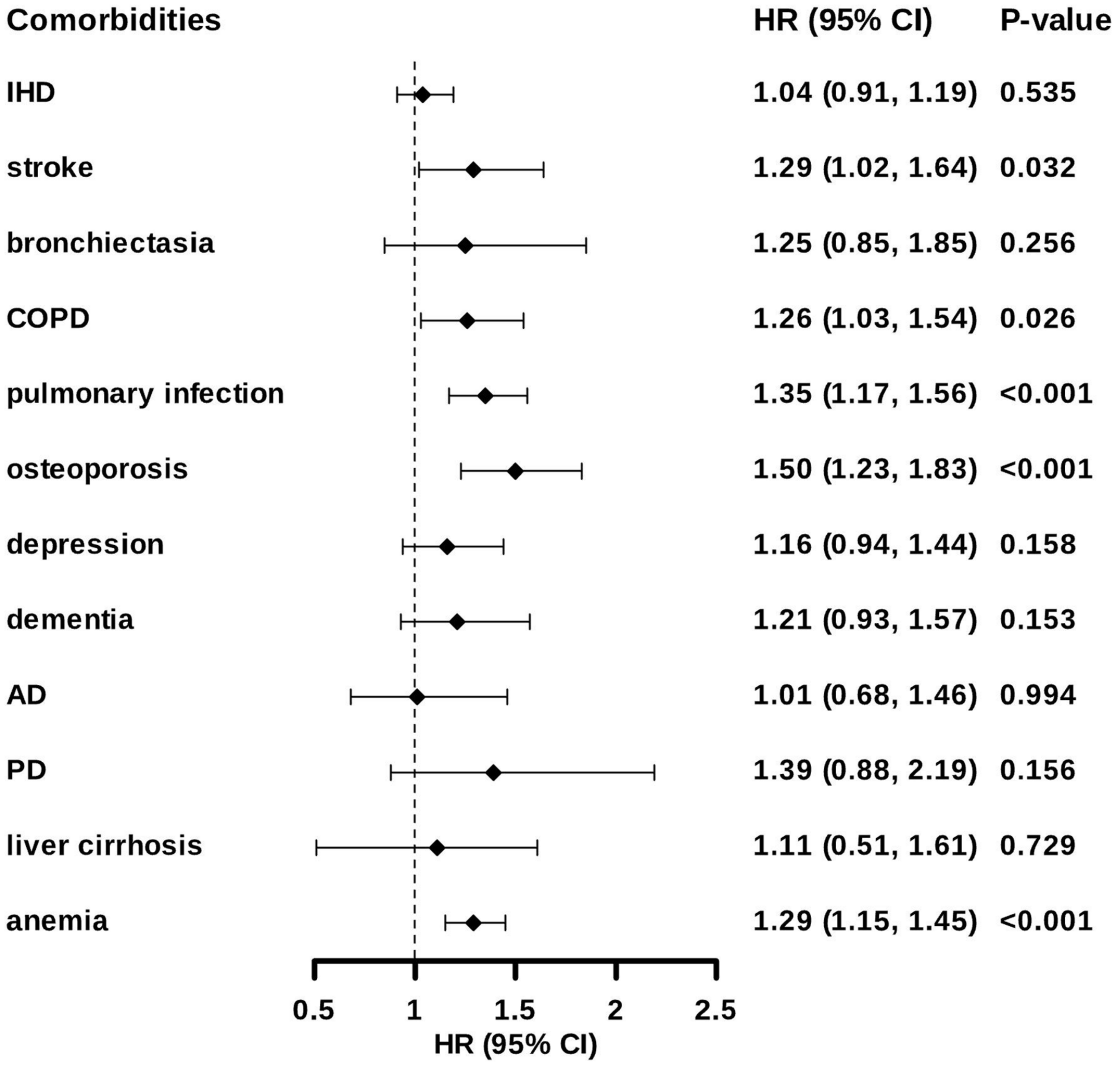
Association between sarcopenia and post-CKD comorbidities in people with CKD. Data are presented as hazard ratios (HRs) and 95% confidence interval (CI). Model was adjusted for age, sex, BMI, ethnicity, education, income, deprivation index, physical activity, smoking status, alcohol intake, DDS, hypertension and diabetes. Abbreviations: HR, hazard ratio; CI, confidence interval; IHD, ischemic heart disease; COPD, chronic obstructive pulmonary disease; AD, Alzheimer’s disease; PD, Parkinson’s disease.

We further examined individual outcomes. The strongest association was observed with osteoporosis (Figure 3). After multivariable adjustment, sarcopenia conferred a 50% increase in osteoporosis risk (95% CI: 1.23, 1.83). Elevated risks of pulmonary infection (HR: 1.35; 95% CI: 1.17, 1.56), anemia (HR: 1.29; 95% CI: 1.15, 1.45), stroke (HR: 1.29; 95% CI: 1.02, 1.64), and COPD (HR: 1.26; 95% CI: 1.03, 1.54) were also observed (Figure 3; Supplementary Table 1). The results of component-level analyses were consistent: low muscle strength, low muscle mass, and slow gait each predicted the highest risk of three or more comorbidities, with HRs ranging from 1.50 to 1.64 (Supplementary Table 2).

### 3.3 Association between handgrip strength and post-CKD comorbidities

**Figure 4:**
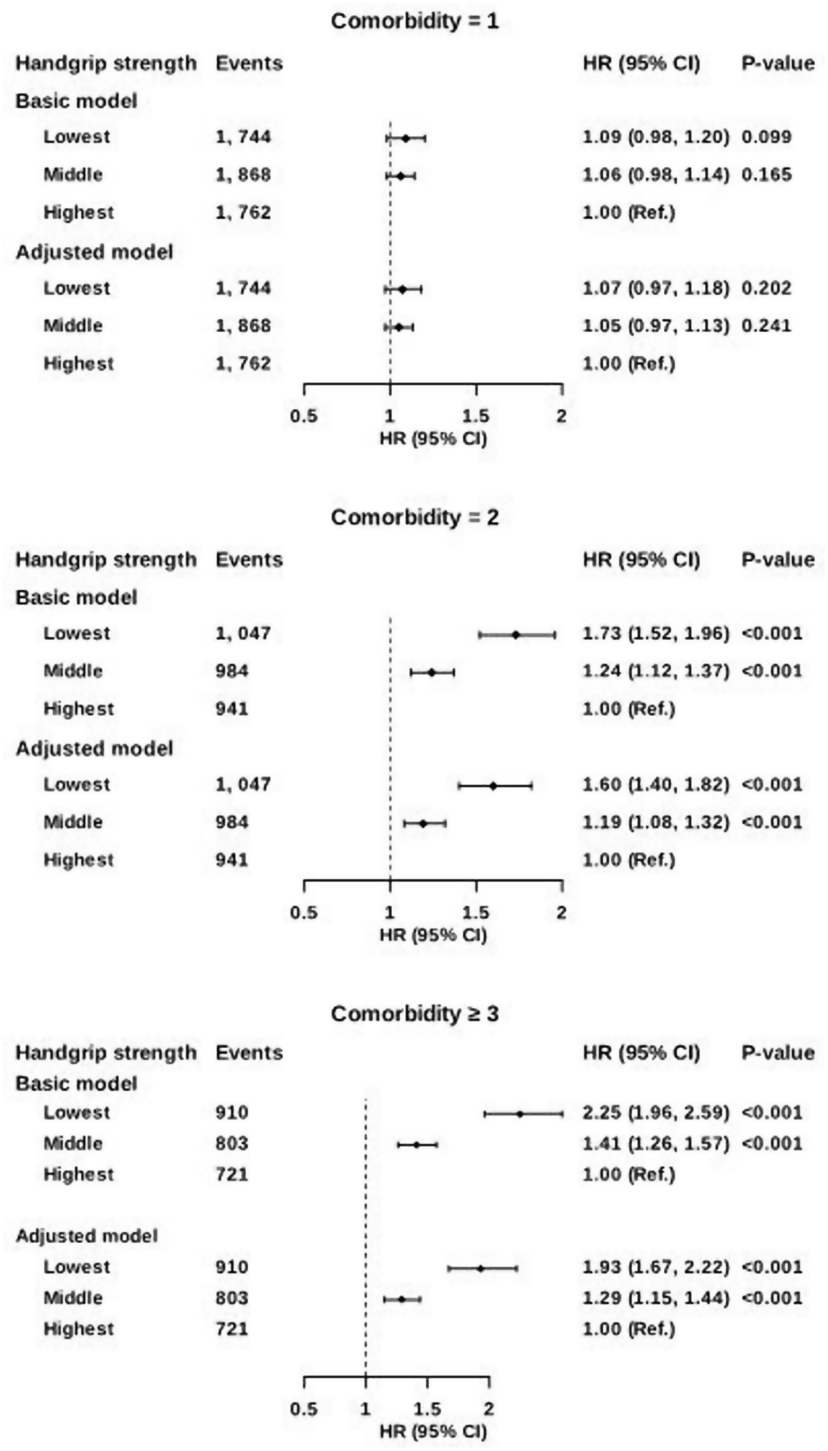
Association between handgrip strength and post-CKD comorbidities. Data are presented as hazard ratios (HRs) and 95% confidence interval (CI). Comorbidities included ischemic heart disease, stroke, bronchiectasia, COPD, pulmonary infection, osteoporosis, depression, dementia, AD, PD, liver cirrhosis and anemia. Basic model was adjusted for age and sex. Adjusted model was additionally adjusted for BMI, ethnicity, education, income, deprivation index, physical activity, smoking status, alcohol intake, DDS, hypertension and diabetes.

Using tertiles, participants in the lowest tertile had the highest risks of one, two, and three or more comorbidities (HRs: 1.07, 1.60, and 1.93, respectively), with the strongest effect observed for three or more comorbidities (HR: 1.93; 95% CI: 1.76, 2.12); this result implies that those in the lowest tertile have a risk of complex multimorbidity nearly twice as high as that in the highest tertile (Figure 4). Penalized cubic-spline analyses showed a monotonic inverse exposure–response between grip strength and the risk of comorbidities (Supplementary Figure 2).

When the lowest and highest handgrip tertiles were compared, the strongest disease-specific associations were seen with Parkinson’s disease (HR: 2.01; 95% CI: 1.33, 3.04), liver cirrhosis (HR: 1.90; 95% CI: 1.27, 2.86), pulmonary infection (HR: 1.71; 95% CI: 1.51, 1.94), and dementia (HR: 1.65; 95% CI: 1.29, 2.12). Notably, osteoporosis showed a pronounced gradient, with the lowest handgrip tertile showing a 77% higher risk (relative to the highest tertile) in the basic model (HR: 1.77; 95% CI: 1.34, 2.32) and a 62% higher risk after multivariable adjustment (HR: 1.62; 95% CI: 1.23, 2.14). These patterns were consistent across cardiovascular, respiratory, musculoskeletal, neuropsychiatric, hepatic, and hematologic comorbidities, with the highest and lowest risks observed in the lowest and highest tertiles, respectively. Across all prespecified conditions, the risks followed a similar graded pattern (Supplementary Table 3).

### 3.4 PAF and PIF results

PAF analysis revealed that sarcopenia accounted for 5.21% (95% CI: 4.68, 5.79), 13.72% (95% CI: 11.83, 15.92), and 23.46% (95% CI: 20.12, 27.35) of the risk of developing one, two, or three or more comorbidities, respectively. In the lowest handgrip tertile, sarcopenia accounted for 28.53% (95% CI: 22.34, 34.37) of the risk of three or more comorbidities, exceeding the corresponding contributions for one or two comorbidities. Improvements in handgrip strength would prevent a larger share of cases as the number of comorbidity increases: PIFs were 12.11% (95% CI: 5.35, 18.90) for one comorbidity, 16.30% (95% CI: 12.75, 19.00) for two, and 22.01% (95% CI: 19.15, 24.16) for three or more under the comprehensive improvement scenario that shifts the lowest and middle handgrip tertiles to the middle and highest tertiles. In a more conservative scenario that shifted only the lowest to the middle tertile, the PIFs were 6.67% (95% CI: 6.13, 7.20) for one comorbidity, 11.19% (95% CI: 10.33, 11.43) for two, and 15.08% (95% CI: 15.00, 15.16) for three or more (Supplementary Table 4).

### 3.5 Stratified analyses results

Stratified analyses results were relatively accordant by sex, age, BMI, physical activity, smoking, alcohol, and dietary diversity (Supplementary Figures 3–5). For most characteristics, we did not find statistically significant effect modifications. But intriguingly, significant interactions between sarcopenia and smoking for one and two comorbidities. Among previous/current smokers, sarcopenia was associated with approximately 24 and 43% higher risks of developing one and two comorbidities, respectively (*p* for interaction = 0.021 and 0.026). In addition, the associations between sarcopenia and the risk of developing one or two comorbidities appeared to be stronger among adults aged ≥65 years (HR: 1.31; 95% CI: 1.07, 1.62; *p* for interaction = 0.009) and among those with low levels of physical activity (HR: 1.25; 95% CI: 1.05, 1.49; *p* for interaction = 0.036). For the development of three or more comorbidities, individuals with a BMI < 25 kg/m^2^ had an increased risk (HR 1.42; 95% CI 1.20–1.69; *p* for interaction = 0.036).

### 3.6 Sensitivity analyses results

All four sensitivity analyses produced results consistent with the main findings (Supplementary Table 5). Specifically, recalculating the eGFR using the 2012 CKD-EPI equation yielded HRs nearly identical to those in the primary analysis (e.g., HR for three or more comorbidities: 1.33; 95% CI: 1.14–1.55). Excluding participants with incomplete covariate data also had a minimal impact on the results (HR for three or more comorbidities: 1.39; 95% CI: 0.94–1.68), as did excluding individuals with any CKD diagnosis in the 2-year look-back period before the baseline assessment (HR for one comorbidity: 1.21; 95% CI: 1.03–1.45). The standardized mean differences between participants with and without sarcopenia were assessed using inverse probability weighting (IPW) (Supplementary Table 6). The results revealed no significant differences in the covariates and did not change substantially when an IPW-weighted Cox model was used (HR for two comorbid conditions: 1.27; 95% CI: 1.10–1.48).

## 4 Discussion

### 4.1 Summary of main findings

Based on a large cohort, this study examined the association between sarcopenia and post-CKD comorbidities, in which sarcopenia was diagnosed according to the EWGSOP2 criteria that focus on three dimensions: muscle strength, muscle mass, and physical performance (7). We observed that sarcopenia was associated with an increased risk of post-CKD comorbidities, with the greatest relative increase for three or more comorbidities. Osteoporosis showed the strongest association with sarcopenia. Handgrip strength was found to be inversely associated with multimorbidity, with a clear exposure–response relationship. At the population level, approximately a quarter of cases of complex multimorbidity were attributable to sarcopenia (PAF = 23.46%), and a feasible improvement scenario involving an upward shift in handgrip strength by one tertile corresponded to a similar preventable burden (PIF = 22.01%).

### 4.2 Interpretation of the findings

Our findings align with and extend those of prior CKD and UK Biobank studies linking low muscle function to adverse outcomes (10, 26, 31). However, we have moved beyond single endpoints by quantifying multimorbidity accumulation as a clinically relevant trajectory (4). The prominence of osteoporosis in our results is consistent with the bone–muscle–kidney axis and the emerging concept of osteosarcopenia in CKD (11, 14, 32), as well as with evidence on vitamin D status, bone microarchitecture, insulin resistance, and metabolic changes in early-stage CKD that influence bone–muscle crosstalk (33, 34). We also demonstrated a clear inverse exposure–response relationship between handgrip strength and CKD comorbidity risk and translate individual-level associations into population impact via PAF and PIF analyses (29, 30), an approach infrequently reported in this field.

Based on this analysis, we propose a unified dual-pathway model—functional decline and systemic dysregulation—through which sarcopenia accelerates comorbidity accumulation in CKD. Along the functional pathway, lower strength, reduced performance, and physical inactivity are associated with bone loss or osteoporosis, falls, and metabolic impairment (e.g., insulin resistance) (14, 32, 34). Along the systemic dysregulation pathway, mineral–bone disorders and endocrine–metabolic disturbances (e.g., vitamin D deficiency, insulin resistance, alterations of hormonal axes), together with inflammatory and metabolic processes (e.g., metabolic acidosis and uremic toxins) (11–13,32–34), may further deplete musculoskeletal reserves. Consistent with prior clinical observations, this synthesis accords with reported links among sarcopenia, anemia, disability, and cognition (15) and with CKD-related muscle energy stress (35). In addition, the bone–muscle–kidney axis provides a mechanistic basis for the prominent association between sarcopenia and osteoporosis (11, 32). Accordingly, this framework explains the prominence of osteoporosis and is consistent with the observed exposure–response gradient and PAF/PIF estimates described above, thereby positioning muscle health as a tractable clinical target in CKD.

Biological and behavioral considerations may explain the observed subgroup patterns. In older adults, anabolic resistance and skeletal muscle energy stress provide a foundation for increased comorbidity (3, 15, 35). A low BMI plausibly reflects undernutrition and low muscle reserves (26, 34). A low level of physical activity removes an anabolic stimulus and is common among patients with CKD (36, 37). Lifestyle exposures such as smoking may exacerbate inflammatory and metabolic stress in patients with CKD (13). Clinically, older adults, low-BMI and low level of physical activity patients should receive prioritized strength screening, early bone health assessment, and targeted lifestyle therapy with support for smoking cessation.

Handgrip strength is a pragmatic bedside metric endorsed in definitions of sarcopenia (7). It has been shown to improve cardiovascular risk prediction in large cohorts (38) and to attenuate diabetes-associated excess risks of cardiovascular disease and mortality in the UK Biobank cohort (39). In the current study, the graded pattern observed across handgrip tertiles provides support for risk-stratified bone health assessment, infection prevention, and targeted rehabilitation. From a population perspective, PAF and PIF indicate that feasible gains in handgrip strength could reduce complex multimorbidity. Implementation efforts could leverage low-cost strength training, nutrition (including protein and vitamin D), and activity programs, thereby addressing the potential contributions of low activity across CKD stages (36, 37) In summary, integrating handgrip strength screening with tiered interventions may help reduce the comorbidity burden among patients with CKD.

### 4.3 Strengths and limitations

This study provided new insights into the association between sarcopenia and the risk of various CKD comorbidities in CKD patients. A key strength of this study is the translation of associations into public health impacts by using PAFs and PIFs, which are seldom applied in the literature on CKD and sarcopenia; these values provide actionable estimates of preventable multimorbidity with plausible improvements in handgrip strength. Additionally, leveraging the large, well-characterized UK Biobank cohort enabled rigorous control of demographic, socioeconomic, lifestyle, and clinical confounders (40), while robust CKD ascertainment included the race-free 2021 eGFRcr-cys, which minimizes differences between Black and non-Black participants (19).

Several limitations should also be noted. First, the ALMI was estimated using an equation from a previous study that used appendicular fat-free mass values. Although dual-energy X-ray absorptiometry is the most commonly used method to measure muscle mass, only 5,000 participants in the UK Biobank study were assessed using this approach (31), which may have introduced bias. Second, as the UK Biobank study did not include data from tests such as the chair stand test, gait speed test, or the Short Physical Performance Battery as proposed by the EWGSOP2, we relied on self-reported walking speed as a proxy to identify patients with severe sarcopenia and found it to be strongly associated with different health outcomes (38). This approach may have introduced bias. Third, as more than two measurements of serum creatinine and cystatin C were available for only 17,841 patients, and those measurements were taken more than 2 years apart, we relied on a single eGFR calculated using the baseline serum creatinine and cystatin C measurements for CKD diagnosis, rather than continuous measurements, which might have resulted in misclassification bias. Fourth, our research focused on 12 comorbidities associated with CKD, whereas other diseases, such as thyroid diseases and some cancers, were not recorded because of sample size limitations. Fifth, we could not account for the participants’ dialysis status because reliable dialysis data were unavailable at baseline. Given the known differences in muscle composition and function between patients with dialysis- and non-dialysis-treated CKD, we cannot exclude the possibility of residual confounding by dialysis. Sixth, reliance on single-time-point assessments (e.g., baseline handgrip strength) and self-reported measures for covariates and outcomes may have introduced some misclassification. Seventh, we only accounted for baseline renal function and were unable to incorporate dynamic changes in kidney function over follow-up, which may have resulted in residual confounding and underestimation of the complex bidirectional relationship between CKD progression and sarcopenia. Finally, we cannot rule out healthy responder bias in the UK Biobank data; in other words, the prevalence of sarcopenia among participants with CKD may have been lower than that in the general population (41). We used IPW to adjust the baseline differences and increase the comparability of HRs across groups (42); this is included as a sensitivity analysis in the Supplementary material.

## 5 Conclusion

In this large cohort of CKD patients, sarcopenia and lower handgrip strength were found to be independently associated with an increased risk of subsequent comorbidity, most notably osteoporosis. PAF and PIF estimates identified handgrip strength as a modifiable clinical target with the potential to lower the burden of comorbidity. Accordingly, CKD care should incorporate routine handgrip strength assessment and strength-preserving interventions. These strategies warrant prospective evaluation.

## Data Availability

The data analyzed in this study is subject to the following licenses/restrictions: individual-level data from the UK Biobank are not publicly available due to their policy, but the data will be made available after the application of the UK Biobank. Requests to access these datasets should be directed to https://www.ukbiobank.ac.uk/.
