# Supplementary material for: Sarcopenia associated with increased risk of comorbidities among individuals with chronic kidney disease: insights from a prospective cohort
Source: Front Public Health. 2025 Dec 16;13:1672639. doi: 10.3389/fpubh.2025.1672639 (PMC12747979; doi:10.3389/fpubh.2025.1672639)
Supplement: Supplementary file 1 [file Table_1.DOCX]

Supplementary Material

# Supplementary Data

**1.1 ICD-10 Codes for Comorbidity Definitions**

Ischemic heart disease (I20–I25); stroke (I60–I64); bronchiectasis (J47); chronic obstructive pulmonary disease (J44); pulmonary infection, including viral and bacterial pneumonia (J12–J18); osteoporosis (M80–M82); depression (F32–F33); dementia (F00–F03); Alzheimer’s disease (G30); Parkinson’s disease (G20); liver cirrhosis (K74); and anemia (D50, D50.0, D50.1, D50.9, D51, D51.0, D51.1, D51.9, D52, D52.0, D52.9, D53, D55–D64, D64.9). Comorbidity counts were derived from these codes harmonized across linked electronic health records and participant self‑reports.

**1.2 Diagnosis of sarcopenia**

Handgrip strength: A Jamar J00105 hydraulic hand dynamometer was used to measure hand grip strength, and low muscle strength was defined as <27 kg among men and <16kg among women.

ALMI: ALMI was calculated by dividing appendicular lean mass (ALM) by height squared, as follows: ALMI (kg/m^2^) = ALM (kg) / Height (m)^2^. Appendicular fat-free mass (AFFM) was assessed using bioelectrical impedance analysis (BIA, Tanita BC 418ma Body Fat Analyzer), which has been widely used to assess body fat and fat-free mass (1), and standing height was measured by a Seca 202 device. An equation based on 4350 participants in UK Biobank who underwent a dual-energy x-ray absorptiometry (DXA) in a later assessment was used to calculate ALM (2): ALM (kg) = 0.958 × AFFM (kg) + sex × 0.166 – 0.308, where sex is coded as 1 for men and 0 for women. Low muscle mass was defined as ALMI < 7.0 kg/m^2^ among men and <5.5 kg/m^2^ among women.

Walking pace: Walking pace was self-reported and classified into slow (<3 miles per hour), steady/average (3-4 miles per hour), and fast (>4 miles per hour).

**1.3 Sensitivity analyses**

We conducted a series of sensitivity analyses to assess the robustness of our findings. a) We recalculated the estimated glomerular filtration rate (eGFR) using the Chronic Kidney Disease Epidemiology Collaborative 2012 equation (3), one of the most commonly used formulas for estimating the eGFR. b) We excluded participants who had incomplete data on covariates. c) We used inverse probability of treatment weighting (IPW) to adjust for baseline differences between those with and those without sarcopenia, calculating treatment probabilities using logistic regression with previously described covariates (4). We used truncated weights to deal with possible extreme weights (5). Specifically, we replaced the weights of observations in the <0.5% (>99.5%) quartile with the 0.5% (99.5%) thresholds. An IPW-weighted Cox model was constructed to examine the association between sarcopenia and the risk of comorbidities in CKD patients. d) We excluded participants with CKD diagnoses during a 2-year look-back period.

# Supplementary Figures and Tables

## Supplementary Figures


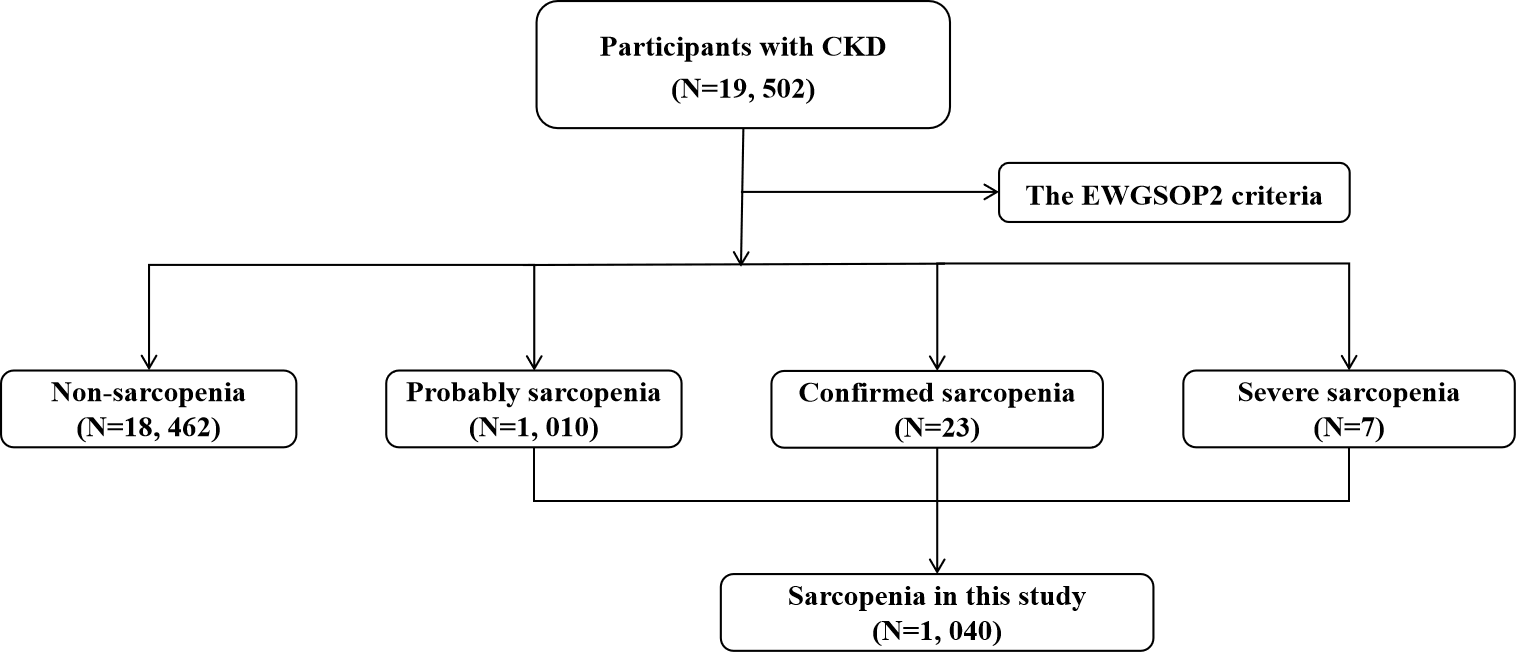


**Supplementary Figure 1. Flowchart of sarcopenia participants in CKD individuals.**

**
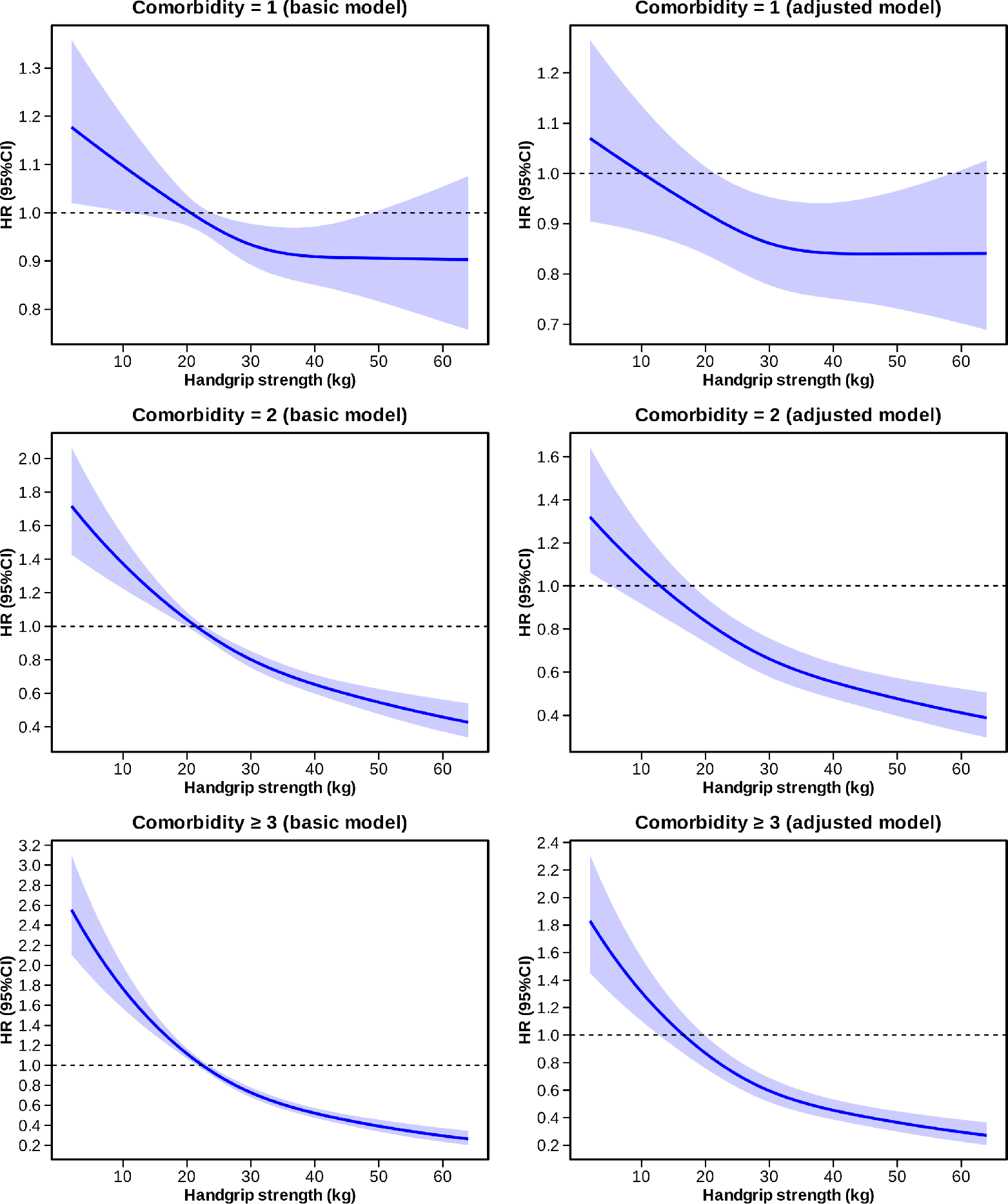
**

**Supplementary Figure 2. Exposure-response associations between handgrip strength and post-CKD comorbidities.**

Data are presented as hazard ratios (HRs) and 95% confidence interval (CI). Handgrip strength was expressed in absolute terms. Associations were modelled by penalized cubic splines with 3 knots. Comorbidities included ischemic heart disease, stroke, bronchiectasia, chronic obstructive pulmonary disease, pulmonary infection, osteoporosis, depression, dementia, Alzheimer's disease, Parkinson's disease, liver cirrhosis and anemia. Basic model was adjusted for age and sex. Adjusted model was additionally adjusted for BMI, ethnicity, education, income, deprivation index, physical activity, smoking status, alcohol intake, DDS, hypertension and diabetes.


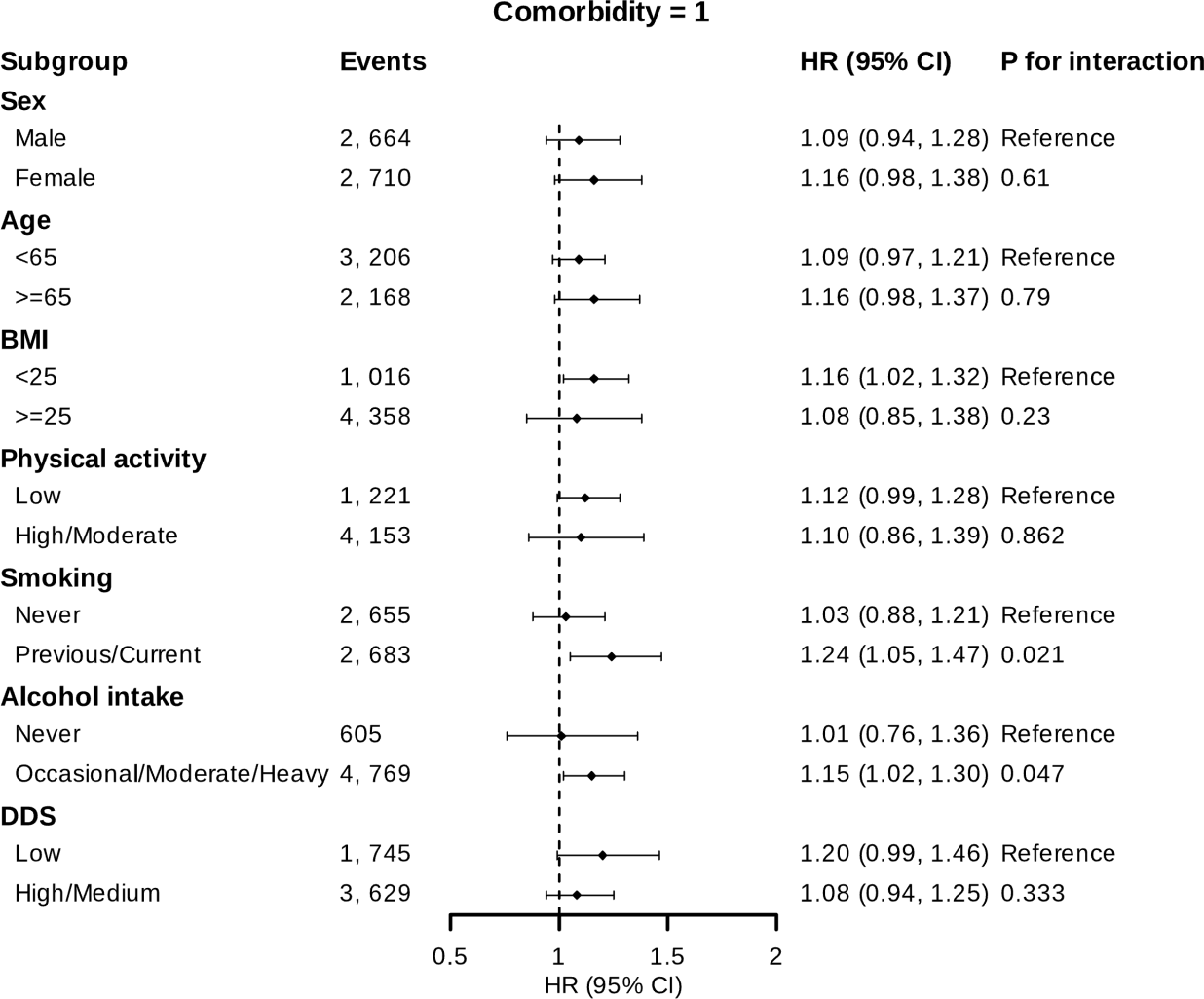


**Supplementary Figure 3. Stratified analyses of association between sarcopenia and post-CKD comorbidities (n=1).**

Data are presented as hazard ratios (HRs) and 95% confidence interval (CI). Comorbidities included ischemic heart disease, stroke, bronchiectasia, chronic obstructive pulmonary disease, pulmonary infection, osteoporosis, depression, dementia, Alzheimer's disease, Parkinson's disease, liver cirrhosis and anemia. It was adjusted for age, sex, BMI, ethnicity, education, income, deprivation index, physical activity, smoking status, alcohol intake, DDS, hypertension and diabetes.


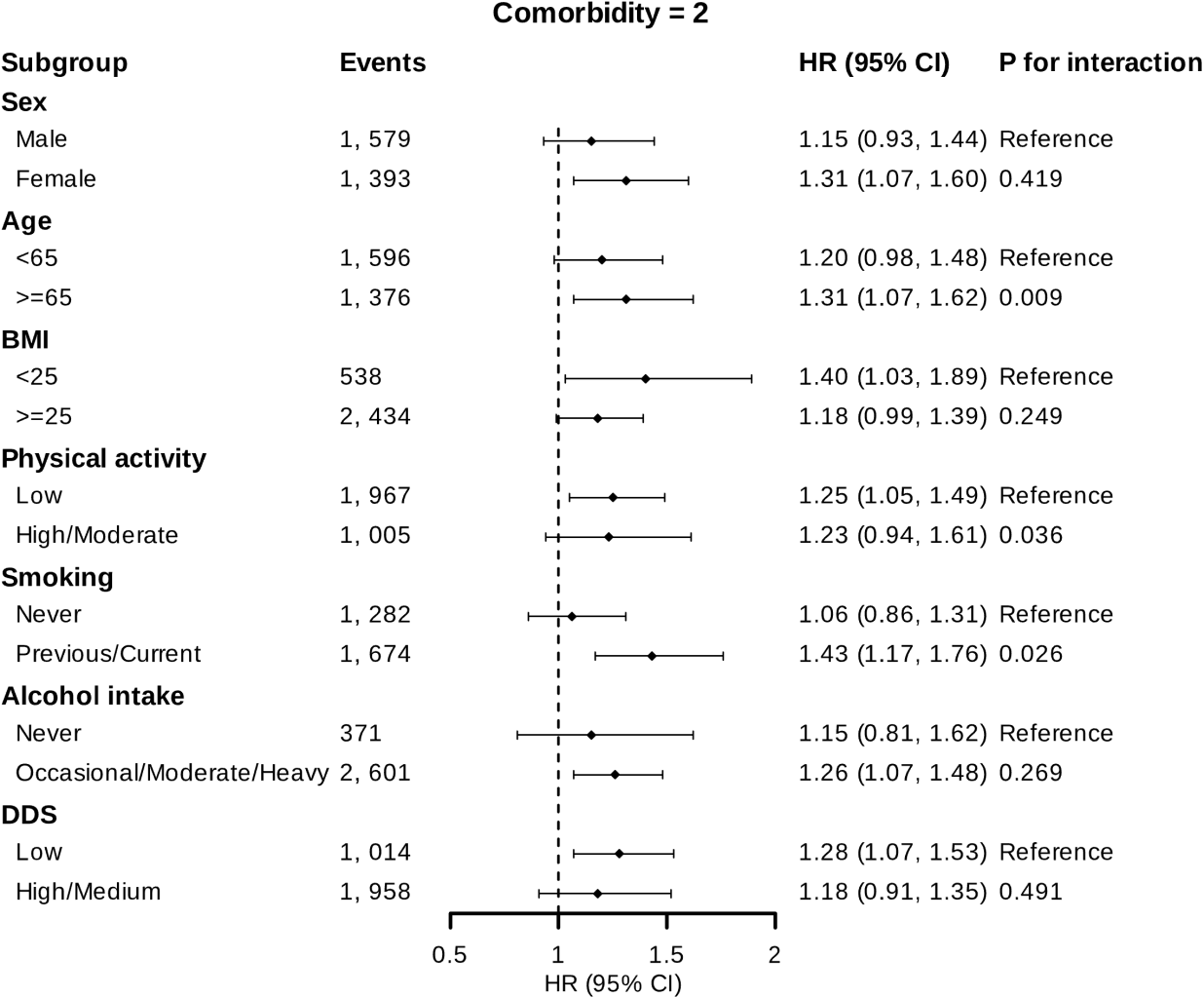


**Supplementary Figure 4. Stratified analyses of association between sarcopenia and post-CKD comorbidities (n=2).**

Data are presented as hazard ratios (HRs) and 95% confidence interval (CI). Comorbidities included ischemic heart disease, stroke, bronchiectasia, chronic obstructive pulmonary disease, pulmonary infection, osteoporosis, depression, dementia, Alzheimer's disease, Parkinson's disease, liver cirrhosis and anemia. It was adjusted for age, sex, BMI, ethnicity, education, income, deprivation index, physical activity, smoking status, alcohol intake, DDS, hypertension and diabetes.


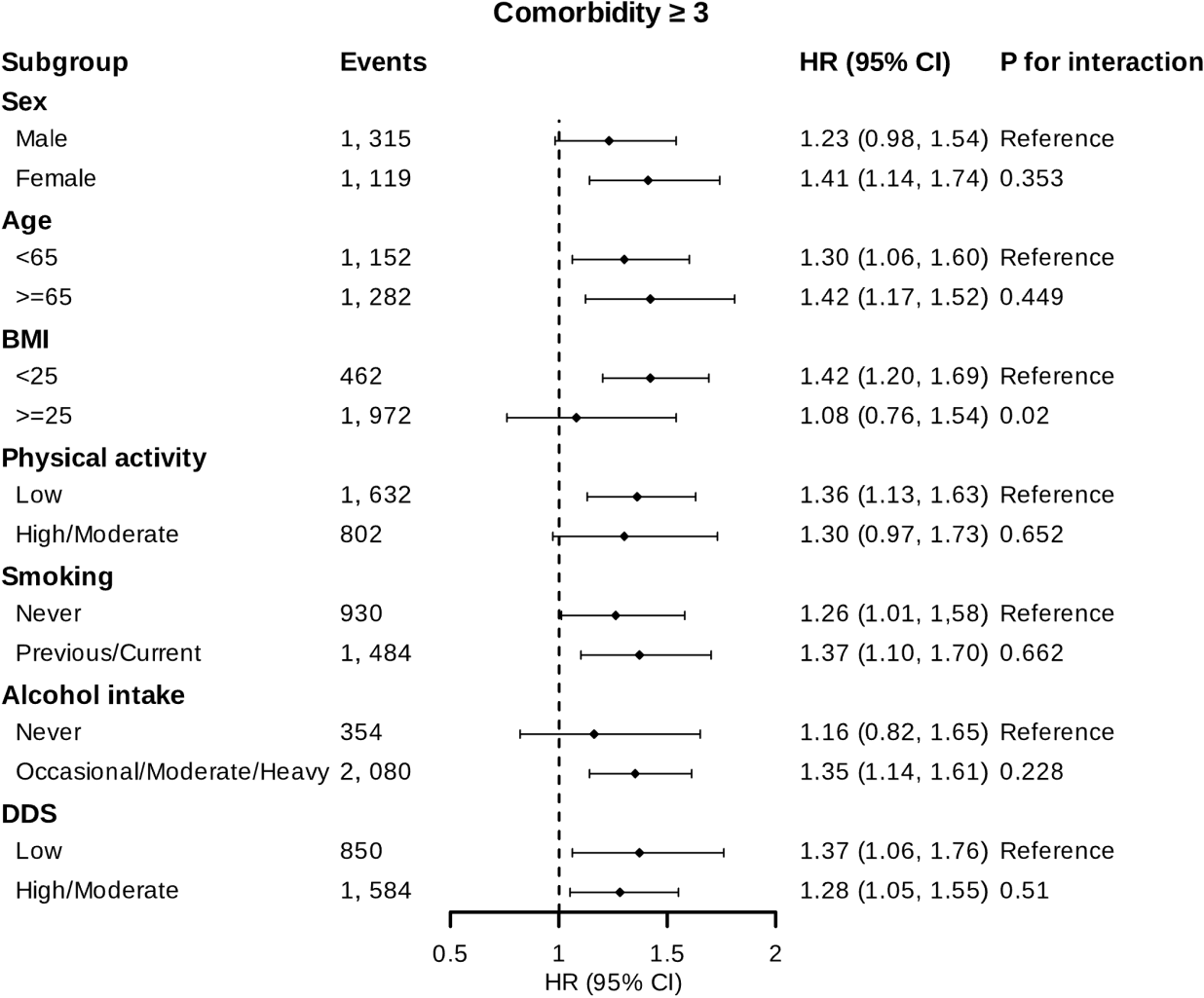


**Supplementary Figure 5. Stratified analyses of association between sarcopenia and post-CKD comorbidities (n≥3).**

Data are presented as hazard ratios (HRs) and 95% confidence interval (CI). Comorbidities included ischemic heart disease, stroke, bronchiectasia, chronic obstructive pulmonary disease, pulmonary infection, osteoporosis, depression, dementia, Alzheimer's disease, Parkinson's disease, liver cirrhosis and anemia. It was adjusted for age, sex, BMI, ethnicity, education, income, deprivation index, physical activity, smoking status, alcohol intake, DDS, hypertension and diabetes.

## Supplementary **Table**s

**Supplementary Table 1. Association between sarcopenia and post-CKD comorbidities in people with CKD**

|  | **Basic model^a^** | | **Adjusted model^b^** | |
| --- | --- | --- | --- | --- |
|  | **HR (95% CI)** | ***P*-value** | **HR (95% CI)** | ***P*-value** |
| IHD | 1.08 (0.95, 1.23) | 0.251 | 1.04 (0.91, 1.19) | 0.535 |
| stroke | 1.23 (0.98, 1.55) | 0.071 | 1.29 (1.02, 1.64) | 0.032 |
| bronchiectasia | 1.25 (0.85, 1.82) | 0.255 | 1.25 (0.85, 1.85) | 0.256 |
| COPD | 1.26 (1.04, 1.53) | 0.022 | 1.26 (1.03, 1.54) | 0.026 |
| pulmonary infection | 1.37 (1.19, 1.57) | <0.001 | 1.35 (1.17, 1.56) | <0.001 |
| osteoporosis | 1.59 (1.31, 1.93) | <0.001 | 1.50 (1.23, 1.83) | <0.001 |
| depression | 1.20 (0.98, 1.47) | 0.076 | 1.16 (0.94, 1.44) | 0.158 |
| dementia | 1.29 (1.01, 1.65) | 0.044 | 1.21 (0.93, 1.57) | 0.153 |
| AD | 1.23 (0.87, 1.74) | 0.235 | 1.01 (0.68, 1.46) | 0.994 |
| PD | 1.50 (0.97, 2.32) | 0.068 | 1.39 (0.88, 2.19) | 0.156 |
| liver cirrhosis | 1.13 (0.57, 1.69) | 0.937 | 1.11 (0.51, 1.61) | 0.729 |
| anemia | 1.38 (1.23, 1.55) | <0.001 | 1.29 (1.15, 1.45) | <0.001 |
| Abbreviations: HR, hazard ratio; CI, confidence interval; IHD, ischemic heart disease; COPD, chronic obstructive pulmonary disease; AD, Alzheimer's disease; PD, Parkinson's disease.  a. Basic model: adjusted for age and sex.  b. Adjusted model: adjusted for age, sex, BMI, ethnicity, education, income, deprivation index, physical activity, smoking status, alcohol intake, dietary diversity score (DDS), hypertension and diabetes.  (**p*<.05) | | | | |

**Supplementary Table 2. Associations between low muscle strength, low muscle mass, low physical performance and post-CKD comorbidities in people with CKD**

|  | **Comorbidity = 1** | | **Comorbidity = 2** | | **Comorbidity ≥ 3** | |
| --- | --- | --- | --- | --- | --- | --- |
|  | **HR (95% CI)** | ***P* value** | **HR (95% CI)** | ***P* value** | **HR (95% CI)** | ***P* value** |
| **Basic model^a^** |  |  |  |  |  |  |
| low muscle strength | 1.14 (1.03, 1.25) | 0.009 | 1.42 (1.26, 1.59) | <0.001 | 1.68 (1.48, 1.90) | <0.001 |
| low muscle mass | 1.10 (0.87, 1.39) | 0.417 | 1.15 (0.89, 1.50) | 0.294 | 1.75 (1.35, 2.27) | <0.001 |
| low physical performance | 1.02 (0.94, 1.10) | 0.634 | 1.45 (1.33, 1.60) | <0.001 | 1.84 (1.42, 2.26) | <0.001 |
| **Adjusted model^b^** |  |  |  |  |  |  |
| low muscle strength | 1.12 (1.02, 1.24) | 0.022 | 1.34 (1.19, 1.51) | <0.001 | 1.50 (1.33, 1.70) | <0.001 |
| low muscle mass | 1.15 (0.90, 1.47) | 0.262 | 1.18 (0.93, 1.53) | 0.194 | 1.55 (1.17, 2.06) | 0.002 |
| low physical performance | 1.01 (0.92, 1.08) | 0.912 | 1.25 (1.13, 1.38) | <0.001 | 1.64 (1.48, 1.81) | <0.001 |
| Abbreviations: HR, hazard ratio; CI, confidence interval.  Comorbidities included ischemic heart disease, stroke, bronchiectasia, chronic obstructive pulmonary disease, pulmonary infection, osteoporosis, depression, dementia, Alzheimer's disease, Parkinson's disease, liver cirrhosis and anemia.   1. Basic model: adjusted for age and sex. 2. Adjusted model: adjusted for age, sex, BMI, ethnicity, education, income, deprivation index, physical activity, smoking status, alcohol intake, dietary diversity score (DDS), hypertension and diabetes.   (**p*<.05) | | | | | | |

**Supplementary Table 3. Association between handgrip strength and and post-CKD comorbidities in people with CKD**

| **Handgrip strength** | **Lowest** | | **Middle** | | **Highest** | |
| --- | --- | --- | --- | --- | --- | --- |
|  | **HR (95% CI)** | **P value** | **HR (95% CI)** | **P value** | **HR (95% CI)** | |
| **Basic model^a^** |  |  |  |  |  | |
| IHD | 1.49 (1.34, 1.66) | <0.001 | 1.23 (1.14, 1.33) | <0.001 | 1.00 (Ref.) | |
| stroke | 1.51 (1.24, 1.83) | <0.001 | 1.21 (1.04, 1.40) | 0.013 | 1.00 (Ref.) | |
| bronchiectasia | 1.53 (1.08, 2.16) | 0.016 | 1.19 (0.90, 1.57) | 0.231 | 1.00 (Ref.) | |
| COPD | 1.63 (1.37, 1.93) | <0.001 | 1.20 (1.05, 1.37) | 0.006 | 1.00 (Ref.) | |
| pulmonary infection | 1.84 (1.64, 2.08) | <0.001 | 1.27 (1.16, 1.40) | <0.001 | 1.00 (Ref.) | |
| osteoporosis | 1.77 (1.34, 2.32) | <0.001 | 1.18 (0.91, 1.52) | 0.211 | 1.00 (Ref.) | |
| depression | 1.78 (1.47, 2.14) | <0.001 | 1.30 (1.11, 1.53) | 0.001 | 1.00 (Ref.) | |
| dementia | 1.84 (1.45, 2.33) | <0.001 | 1.24 (1.02, 1.50) | 0.028 | 1.00 (Ref.) | |
| AD | 1.51 (1.06, 2.14) | 0.021 | 1.36 (1.04, 1.80) | 0.027 | 1.00 (Ref.) | |
| PD | 2.00 (1.34, 2.99) | <0.001 | 1.45 (1.06, 1.99) | 0.019 | 1.00 (Ref.) | |
| liver cirrhosis | 2.15 (1.44, 3.19) | <0.001 | 1.45 (1.07, 1.99) | 0.018 | 1.00 (Ref.) | |
| anemia | 1.77 (1.60, 1.96) | <0.001 | 1.31 (1.20, 1.41) | <0.001 | 1.00 (Ref.) | |
| **Adjusted model^b^** |  |  |  |  |  |  |
| IHD | 1.35 (1.22, 1.51) | <0.001 | 1.18 (1.09, 1.28) | <0.001 | 1.00 (Ref.) | |
| stroke | 1.44 (1.18, 1.77) | <0.001 | 1.17 (1.01, 1.36) | 0.039 | 1.00 (Ref.) | |
| bronchiectasia | 1.44 (1.01, 2.04) | 0.043 | 1.14 (0.86, 1.52) | 0.354 | 1.00 (Ref.) | |
| COPD | 1.46 (1.23, 1.74) | <0.001 | 1.12 (0.98, 1.28) | 0.105 | 1.00 (Ref.) | |
| pulmonary infection | 1.71 (1.51, 1.94) | <0.001 | 1.23 (1.12, 1.36) | <0.001 | 1.00 (Ref.) | |
| osteoporosis | 1.62 (1.23, 2.14) | <0.001 | 1.14 (0.88, 1.48) | 0.323 | 1.00 (Ref.) | |
| depression | 1.68 (1.39, 2.04) | <0.001 | 1.26 (1.07, 1.48) | 0.006 | 1.00 (Ref.) | |
| dementia | 1.65 (1.29, 2.12) | <0.001 | 1.17 (0.97, 1.43) | 0.107 | 1.00 (Ref.) | |
| AD | 1.30 (0.90, 1.86) | 0.157 | 1.33 (1.01, 1.77) | 0.048 | 1.00 (Ref.) | |
| PD | 2.01 (1.33, 3.04) | <0.001 | 1.50 (1.08, 2.07) | 0.015 | 1.00 (Ref.) | |
| liver cirrhosis | 1.90 (1.27, 2.86) | 0.002 | 1.40 (1.02, 1.92) | 0.038 | 1.00 (Ref.) | |
| anemia | 1.55 (1.40, 1.72) | <0.001 | 1.21 (1.11, 1.31) | <0.001 | 1.00 (Ref.) | |
| Abbreviations: HR, hazard ratio; CI, confidence interval; IHD, ischemic heart disease; COPD, chronic obstructive pulmonary disease; AD, Alzheimer's disease; PD, Parkinson's disease.   1. Basic model: adjusted for age and sex.   b. Adjusted model: adjusted for age, sex, BMI, ethnicity, education, income, deprivation index, physical activity, smoking status, alcohol intake, dietary diversity score (DDS), hypertension and diabetes.  (**p*<.05) | | | | | | |

**Supplementary Table 4. Population attributable fractions and potential impact fractions**

|  | **Comorbidity = 1** | **Comorbidity = 2** | **Comorbidity ≥ 3** |
| --- | --- | --- | --- |
| **PAF (95% CI)** |  |  |  |
| Attributable to the lowest tertiles of handgrip strength | 15.96 (20.14, 24.50) | 19.61 (13.98, 25.00) | 28.53 (22.34, 34.37) |
| Attributable to sarcopenia | 5.21 (4.68, 5.79 ) | 13.72 (11.83, 15.92) | 23.46 (20.12, 27.35) |
| **PIF (95% CI)** |  |  |  |
| Moved the lowest tertile to the middle tertile, and the middle tertile to the highest tertile | 12.11 (5.35, 18.9) | 16.30 (12.75, 19.00) | 22.01 (19.15, 24.16) |
| Moved only people in the lowest tertile to the middle tertile | 6.67 (6.13, 7.20) | 11.19 (10.33, 11.43) | 15.08 (15.00, 15.16) |
| Abbreviations: PAF, population attributable fractions; PIF, potential impact fractions.  Comorbidities included ischemic heart disease, stroke, bronchiectasia, chronic obstructive pulmonary disease, pulmonary infection, osteoporosis, depression, dementia, Alzheimer's disease, Parkinson's disease, liver cirrhosis and anemia. Estimated based on HR shown in Figure 3, which adjusted for age, sex, BMI, ethnicity, education, income, deprivation index, physical activity, smoking status, alcohol intake, dietary diversity score (DDS), hypertension and diabetes. | | | |

**Supplementary Table 5. Sensitivity analysis for associations of sarcopenia with comorbidity.**

|  | **Comorbidity = 1** | | **Comorbidity = 2** | | **Comorbidity ≥ 3** | |
| --- | --- | --- | --- | --- | --- | --- |
|  | **HR (95% CI)** | ***P* value** | **HR (95% CI)** | ***P* value** | **HR (95% CI)** | ***P* value** |
| Recalculating eGFR by the CKD Epidemiology Collaborative 2012 equation | 1.12 (1.01, 1.26) | 0.046 | 1.24 (1.07, 1.44) | 0.004 | 1.33 (1.14, 1.55) | <0.001 |
| Excluding participants who had incomplete covariate data | 1.29 (0.91, 1.83) | 0.151 | 1.37 (0.92, 1.82) | 0.266 | 1.39 (0.94, 1.68) | 0.085 |
| Adjusted through inverse probability weighting using predefined covariates | 1.15 (1.05, 1.32) | 0.015 | 1.27 (1.10, 1.48) | 0.002 | 1.33 (1.13, 1.57) | <0.001 |
| Excluding participants with CKD diagnoses during a 2-year look-back period | 1.21 (1.03, 1.45) | 0.029 | 1.29 (1.10, 1.42) | <0.001 | 1.34 (1.12, 1.65) | 0.003 |
| Abbreviations: HR, hazard ratio; CI, confidence interval.  Comorbidities included ischemic heart disease, stroke, bronchiectasia, chronic obstructive pulmonary disease, pulmonary infection, osteoporosis, depression, dementia, Alzheimer's disease, Parkinson's disease, liver cirrhosis and anemia.  Adjusted for age, sex, BMI, ethnicity, education, income, deprivation index, physical activity, smoking status, alcohol intake, DDS, hypertension and diabetes. | | | | | | |

**Supplementary Table 6. Demographic and health characteristics of the participants with or without sarcopenia and the standardized mean difference before and after weighted**

| **Variables** | **Sarcopenic**  **(n=1, 040)** | **Non-Sarcopenic**  **(n=18, 462)** | **Standardized mean difference before IPW^a^** | **Standardized mean difference after IPW^a^** |
| --- | --- | --- | --- | --- |
|  | **Mean ± SD or n (%)** | |  |  |
| Age (years) | 62.8 (5.9) | 61.5 (6.5) | 0.213 | 0.050 |
| Sex |  |  | 0.102 | 0.039 |
| Female | 589 (56.6) | 9, 515 (51.5) |  |  |
| Male | 451 (43.4) | 8, 947 (48.5) |  |  |
| Ethnicity |  |  | 0.243 | 0.039 |
| White | 932 (89.6) | 17, 435 (94.4) |  |  |
| Non-white | 108 (10.4) | 1, 027 (5.6) |  |  |
| BMI, kg/m^2^ |  |  | 0.057 | 0.024 |
| Underweight (<18.5) | 4 (0.4) | 32 (0.2) |  |  |
| Normal (18.5–25.0) | 212 (20.4) | 3, 558 (19.3) |  |  |
| Overweight (25.0–30.0) | 453 (43.6) | 7, 947 (43.0) |  |  |
| Obese (≥30.0) | 371 (35.7) | 6, 925 (37.5) |  |  |
| Annual household income, € |  |  | 0.261 | 0.067 |
| >100, 000 | 14 (1.3) | 334 (1.8) |  |  |
| 52, 000–100, 000 | 58 (5.6) | 1, 761 (9.5) |  |  |
| 31, 000–51, 999 | 126 (12.1) | 3, 212 (17.4) |  |  |
| 18, 000–30, 999 | 240 (23.1) | 4, 532 (24.5) |  |  |
| <18, 000 | 351 (33.8) | 5, 223 (28.3) |  |  |
| Unknown | 251 (24.1) | 3, 400 (18.4) |  |  |
| Education qualifications |  |  | 0.195 | 0.029 |
| College or university degree | 192 (18.5) | 4, 029 (21.8) |  |  |
| A levels/AS levels or equivalent | 89 (8.6) | 1, 619 (8.8) |  |  |
| O levels/GCSEs or equivalent | 190 (18.3) | 3, 978 (21.5) |  |  |
| CSEs or equivalent | 40 (3.8) | 802 (4.3) |  |  |
| NVQ or HND or HNC or equivalent | 73 (7.0) | 1, 478 (8.0) |  |  |
| Other (eg: nursing, teaching) | 62 (6.0) | 1, 135 (6.1) |  |  |
| None of the above | 394 (37.9) | 5, 421 (29.4) |  |  |
| Deprivation index | -0.7 (3.4) | -1.2 (3.1) | 0.163 | 0.017 |
| Smoking status |  |  | 0.126 | 0.004 |
| Never | 577 (55.5) | 9, 340 (50.6) |  |  |
| Previous | 361 (34.7) | 7, 187 (38.9) |  |  |
| Current | 102 (9.8) | 1, 935 (10.5) |  |  |
| Alcohol intake |  |  | 0.201 | 0.028 |
| Never | 170 (16.3) | 1, 975 (10.7) |  |  |
| Occasional | 289 (27.8) | 5, 029 (27.2) |  |  |
| Moderate | 442 (42.5) | 8, 060 (43.7) |  |  |
| Heavy | 139 (13.4) | 3, 398 (18.4) |  |  |
| Physical activity |  |  | 0.088 | 0.045 |
| Low | 232 (22.3) | 4, 140 (22.4) |  |  |
| Moderate | 479 (46.1) | 7, 783 (42.2) |  |  |
| High | 329 (31.6) | 6, 539 (35.4) |  |  |
| DDS level |  |  | 0.037 | 0.016 |
| Low | 355 (34.1) | 5, 984 (32.4) |  |  |
| Medium | 670 (64.4) | 12, 198 (66.1) |  |  |
| High | 15 (1.4) | 280 (1.5) |  |  |
| Hypertension | 712 (68.5) | 12, 606 (68.3) | 0.004 | 0.010 |
| Diabetes | 192 (18.5) | 2, 777 (15.0) | 0.092 | 0.034 |
| Abbreviations: SD = Standard deviation; BMI= Body mass index; DDS= Dietary diversity.  a.Standardized mean difference less than 0.10 is considered good balance.  (*p<.05) | | | | |

**References**

1. Rudnev S, Burns JS, Williams PL, Lee MM, Korrick SA, Denisova T, et al. Comparison of bioimpedance body composition in young adults in the Russian Children’s Study. Clin Nutr ESPEN (2020) 35:153–61. doi: 10.1016/j.clnesp.2019.10.007

2. Dodds RM, Granic A, Robinson SM, Sayer AA. Sarcopenia, long‐term conditions, and multimorbidity: findings from UK Biobank participants. J Cachexia Sarcopenia Muscle (2020) 11:62–68. doi: 10.1002/jcsm.12503

3. Inker LA, Schmid CH, Tighiouart H, Eckfeldt JH, Feldman HI, Greene T, et al. Estimating glomerular filtration rate from serum creatinine and cystatin C. N Engl J Med (2012) 367:20–29. doi: 10.1056/NEJMoa1114248

4. Studenski SA, Peters KW, Alley DE, Cawthon PM, McLean RR, Harris TB, et al. The FNIH Sarcopenia Project: Rationale, study description, conference recommendations, and final estimates. J Gerontol Ser A (2014) 69:547–58. doi: 10.1093/gerona/glu010

5. Cole SR, Hernan MA. Constructing inverse probability weights for marginal structural models. Am J Epidemiol (2008) 168:656–64. doi: 10.1093/aje/kwn164
